# Technical Lignins Antibacterial Effects Against Environmental Mastitis Pathogens Across Various Levels of Bedding Cleanliness In Vitro

**DOI:** 10.3390/molecules30142904

**Published:** 2025-07-09

**Authors:** Godloves M. Oppong, Diana C. Reyes, Zhengxin Ma, Santiago A. Rivera, Marjorie A. Killerby, Diego Zamudio, Anne B. Lichtenwalner, Juan J. Romero

**Affiliations:** 1 Animal and Veterinary Sciences, School of Food and Agriculture, University of Maine, Orono, ME 04469, USA; godloves.oppong@maine.edu (G.M.O.); dcr232@cornell.edu (D.C.R.); santiago.riverareyes@unh.edu (S.A.R.); killerby@wisc.edu (M.A.K.); diego.zamudio@maine.edu (D.Z.); anne.lichtenwalner@maine.edu (A.B.L.); 2 Molecular and Biomedical Sciences, University of Maine, Orono, ME 04469, USA

**Keywords:** antimicrobial activity, bedding conditioner, dairy cattle bedding, technical lignin

## Abstract

This study aimed to evaluate the antibacterial activity of several technical lignins against major environmental bacteria that cause mastitis in dairy cattle. The efficacy of four types of technical lignins against environmental mastitis pathogens was evaluated using MIC and MBC assays. The best candidate, sodium lignosulfonate (NaL-O), was further tested using sawdust bedding substrates. Substrates were prepared in different cleanliness conditions: sawdust only, sawdust plus urine, sawdust plus feces, or sawdust plus a combination of both. The antimicrobial activity of NaL-O against the mixture of environmental mastitis-causing pathogens was determined on days 0, 2, and 6 of incubation. In addition, the components of bedding substrates were analyzed to help understand the dynamics of pathogen loads. In the MIC and MBC assays, NaL-O showed the best antimicrobial performance against all pathogens except *Escherichia coli*. When testing in the bedding substrates, the addition of NaL-O decreased the concentration of *Staphylococcus chromogenes, Streptococcus uberis*, and *Pseudomonas aeruginosa* across all bedding cleanliness levels at d 0, 2, and 6 of incubation. As the incubation time increased, the antimicrobial effect decreased. NaL-O also lowered the counts of *E. coli* and *Klebsiella pneumoniae* across all incubation times, but to a lesser extent. The presence of feces significantly reduced the antibacterial effects of NaL-O for these two bacteria. Among the technical lignins tested, NaL-O showed the broadest antibacterial activity against the mastitis pathogens tested. This study suggests that NaL-O has promising potential as a bedding conditioner to control environmental pathogens on dairies due to its low cost, ready availability, and compatibility with sustainable livestock practices. Combined with bedding cleanliness, bedding conditioner application may play a crucial role in reducing the growth of EM pathogens and subsequent mastitis incidence.

## 1. Introduction

Mastitis is the most prevalent disease affecting lactating dairy cattle [1], causing an estimated loss of over $2 billion/year in the United States alone [2]. This disease has a detrimental impact on cow health [3], milk yield [4], and milk composition [4]. Mastitis consists of inflammation of the mammary gland, and causes a measurable increase in milk somatic cell counts above 200,000 cells/mL in affected dairy cattle [5,6]. This increase is associated with lower reproductive performance and reduced pregnancy rates [7].

Environmental pathogenic microbes [8] are recognized as the major causative agents of mastitis in lactating dairy cattle and are the focus of the dairy industry’s efforts to decrease mastitis incidence [9]. Environmental mastitis pathogens [10] are found in the bedding of lactating dairy cattle and can cause mastitis when the cows’ teats come into contact with these pathogens in the bedding or during milking [11]. Major environmental mastitis-causing bacteria include *Streptococcus* spp. (not *S. agalactiae*), *Staphylococcus* (not *S. aureus*), *Escherichia coli*, *Klebsiella* spp., *Pseudomonas* spp., and *Enterococcus* spp. [12]. Several studies have shown positive correlations between the incidence of mastitis [11] and the abundance of bacteria in bedding and on teat ends of lactating cows [13,14]. Therefore, reducing pathogen presence in bedding can reduce the risk of mastitis on dairy farms [15]. This is especially true for beddings consisting of organic materials such as sawdust, woodchips, and straw (used in ~61% of U.S. dairy farms; [16]), which are more likely to sustain pathogens than are inorganic bedding materials such as sand and lime [15]. Organic beddings provide more favorable conditions and nutrients for pathogens compared to inorganic beddings and, consequently, are more likely to sustain environmental mastitis-causing microbes [14].

It has been shown that the bedding type influences the abundance and growth rate of specific mastitis-causing pathogens [17]. For instance, wood-based bedding can be a major source of *Klebsiella* spp. and is linked to mastitis outbreaks [18]. To lower microbial burden in beddings, the use of bedding conditioners has been suggested, particularly in organic beddings [15]. Bedding conditioners mainly alter the pH of bedding, either reducing [19] or increasing its value [20] to an extent that inhibits the growth of mastitis pathogens [11]. However, the effect of bedding conditioners on bedding pH is temporary and the effects diminish as incubation days increase [11].

The use of technical lignins for antimicrobial purposes has been explored previously, including its potential as hay [21,22] and wood [23] preservatives. Technical lignins are byproducts of the papermaking industry, produced from the extraction and isolation of lignin from wood and other lignocellulosic materials during the pulping process. The physicochemical properties of these technical lignins vary considerably according to the lignin source (i.e., wood type), and the processing conditions [24], thus also affecting their antimicrobial properties. Particularly, lignosulfonates [25] and kraft lignins [26] have been shown to harbor strong antimicrobial activities. For instance, sodium and magnesium lignosulfonates inhibited *Streptococcus uberis*, *Staphylococcus hyicus*, *Escherichia coli*, *Klebsiella pneumoniae*, and *Pseudomonas aeruginosa* at minimum inhibitory concentrations (MIC) as low as 5.8–6.25 mg/mL [27]. Similarly, sodium lignosulfonate was found to exhibit strong antifungal activity against *Candida albicans*, likely through membrane destabilization and metabolic interference [28]. For kraft lignins, Dong et al. [26] reported that corn stover-derived kraft lignin exhibited MICs of 0.5–1 mg/mL against *Bacillus cereus* and *Staphylococcus aureus*. Wang et al. [29] demonstrated that bamboo kraft lignin fractions, enhanced via ethanol fractionation to improve water solubility, significantly inhibited Gram-positive bacteria. The exact mode of action of these technical lignins against microorganisms has not been fully elucidated. However, it is known that they contain high concentrations of phenolic monomers such as ferulic acid, isoeugenol, and p-coumaryl, which have shown to inhibit the growth of bacteria and fungi [30,31]. Reyes and collaborators [32] comprehensively reviewed the antimicrobial effects of different technical lignins and their derivatives, and proposed other possible modes of action.

Each year, about 50 million Mg of technical lignins are produced in paper mill factories worldwide, with only about 2% being commercialized and the rest being burnt as waste [33]. Additionally, technical lignins are non-toxic, non-corrosive, and biodegradable [34], making them an inexpensive, safe, and sustainable potential alternative for the prevention of mastitis in dairy farms. Therefore, this study aimed to evaluate the antibacterial activity of several technical lignins against major environmental bacteria that cause mastitis in dairy cattle. We hypothesized that certain types of technical lignins could hinder the growth of environmental mastitis-causing microbes and, therefore, could be used as a bedding conditioners for lactating dairy cattle.

## 2. Results

### 2.1. MIC and MBC Tests

Among technical lignins, NaL had the lowest MIC across all bacteria strains evaluated, with values ranging from 5.0 (*S. chromogenes* KCJ4610) to 60 mg/mL (*E. coli* 10.0371) (Table 1). Inhibitory activity was consistent across *S. chromogenes* strains (5–6.25 mg/mL) but more variable for *S. uberis* (5.8–10), *P. aeruginosa* (16.6–35), *E. coli* (27.5–60), and *K. pneumoniae* (8.33–15). Based on these results, NaL was more effective against the Gram-positives in the assay. Likewise, NaL showed the highest bactericidal activity against all bacteria strains evaluated, with MBC values ranging from 5.8 (*S. uberis*) to >60.0 mg/mL (*P. aureginosa*). NaL bactericidal activity was consistent against *S. chromogenes* strains (7.5–8.75 mg/mL), but more variable against *S. uberis* (5.8–15), *P. aeruginosa* (20–>60), *E. coli* (30–60), and *K. pneumoniae* (20–40).

MIC values for MgL ranged from 10 mg/mL (*S. chromogenes* KCJ4610 and *K. pneumoniae* KCJ4740) to 60 mg/mL (*P. aeruginosa* KCJ4686). Across strains within a species, MgL MIC was less variable for *S. uberis* (15–23.3 mg/mL), *E. coli* (20–30), and *K. pneumoniae* (10–20) and more so for *P. aeruginosa* (26.6–60) and *S. chromogenes* (10–25). Similarly, MgL MBC values were less variable for *S. uberis* (30–40 mg/mL), *E. coli* (30–56.7), and *K. pneumoniae* (10–20) but more so for *P. aeruginosa* (40–>60) and *S. chromogenes* (12.5–30).

MIC values for AKL were highly variable across all bacteria tested: *S. chromogenes* (2.5–16.6), *S. uberis* (2.5–10), *P. aeruginosa* (50–>60), *E. coli* (>60), and K. pneumoniae (2.5–50). AKL MBC values were more variable than MIC for *S. chromogenes* (5–50), *S. uberis* (2.5–>60), *P. aeruginosa* (>60), *E. coli* (>60), and *K. pneumoniae* (4.37–>60).

WKL had the lowest inhibitory activity across all tested lignins for *K. pneumoniae*, *E. coli*, and *S. uberis* (>60 mg/mL); for *S. chromogenes* (30–>60); and for *P. aeruginosa* (50–>60). WKL bactericidal activity was weak against *K. pneumoniae* (56.7–>60 mg/mL), *S. chromogenes* (60–>60), and >60 for *S. uberis*, *P. aeruginosa*, and *E. coli*.

Overall, NaL had superior antibacterial activity when compared to all tested technical lignins, except against *E. coli*.

### 2.2. Antimicrobial Activity of CND in Bedding Substrates

The main effects of bedding conditioners (CND), bedding cleanliness (CLN), and incubation days (DAY) on response variables measured, along with 2- and 3-way interactions are summarized in Table 2. Compared to CON, the addition of NaL-O decreased *S. chromogenes* KCJ4610 across all bedding cleanliness conditions by 89.3, 82.2, and 62.9%, and reduced *S. uberis* BAA-854 by 95.0, 91.7, and 68.4% at d 0, 2, and 6 of incubation, respectively (CND × DAY; Table 3). As the incubation period was extended, antibacterial activity of NaL-O decreased against these bacteria. The addition of NaL-O to the bedding decreased *E. coli* 10.0371 and *K. pneumoniae* KCJ4749 across all incubation times for SD, SDU, SDF, and SDFU by 51.0, 60.2, 22.4, and 30.9% and 45.1, 53.2, 24.1, and 22.4%, respectively, relative to CON (CND × CLN; Table 4). The presence of feces reduced the antibacterial effects of NaL-O for these two bacterial strains. Within NaL-O treated beddings, SDF had the highest counts of *E. coli* 10.0371 and *K. pneumoniae* KCJ4749, followed by SDFU, SD, and the lowest counts were observed in SDU. The same pattern was observed in CON beddings of the same type. Thus, not only did feces reduce the efficacy of NaL-O, but also elicited a higher count of these two strains for both NaL-O and CON beddings. NaL-O decreased *P. aeruginosa* KCJ4633 counts vs. CON across all bedding types and incubation time combinations to the same extent (1.90 vs. 2.86 ± 0.134 log CFU/fresh g; *p* < 0.001).

We observed the effect of CLN on bedding DM concentration only (Table 2). SD bedding had the highest DM value (75.2 ± 0.05%), followed by SDU (63.2), and the lowest values were observed for both SDF and SDFU ($\overset{-}{x}$ = 44.8). For OM, 2-way interactions included CND × Day and CND × CLN (Table 2). For the CND × CLN, CON, SDU and SD (98.9 and 98.8 ± 0.270% of DM) have the highest values, followed by SDF (98.0); SDFU has the lowest value (97.3; Table 4). Of NaL-O treatments, SD had the highest OM (99.0 ± 0.27% of DM), followed by SDU (98.3), and then SDF and SDFU (96.6 and 96.3). CON had a higher OM concentration vs. NaL-O in SDF (98.0 vs. 96.6 ± 0.27% of DM) and SDFU (97.3 vs. 96.3). There were no differences in OM between CON and NaL-O in SD (99.0 and 98.8 ± 0.27% of DM) and SDU (98.9 and 98.3). The CND × Day effect showed that for day 0, SD and SDU had the highest OM (98.9 and 98.5, respectively), with SDF and SDFU having lowest (97.3 and 96.9, respectively). On day 6, SD and SDU had the highest OM (98.8 and 98.7, respectively), followed by SDF (97.3), and SDFU (96.6) with the lowest OM.

The only variable affected by a 3-way interaction was bedding pH (CND × CLN × DAY; Table 2 and Table 5). For SD bedding, no differences were observed across incubation days for both NaL-O and CON. Moreover, within a given incubation day, there were no differences between CON and NaL-O. In the case of SDU bedding, higher pH values were observed at d 0 for both NaL-O and CON-treated bedding relative to d 2 and 6, which were not different from each other. There was no effect of adding NaL-O to SDU on pH values within a given incubation day. For CON SDF beddings, the highest pH values were observed at d 6, followed by d 2, with the lowest values found at d 0. When NaL-O was added to SDF beddings, the pH at d 6 was higher than d 0, but d 2 values were no different from d 0 and 6. At d 6, NaL-O-treated SDF beddings had a lower pH value relative to their CON counterpart; on all other days, there were no differences. The largest effects of NaL-O application on pH were observed on SDFU beddings. The addition of NaL-O decreased the pH values across d 0, 2, and 6 (0.46, 0.43, 0.89). Thus, NaL-O was important in keeping the pH low at the longest incubation times tested. The pH in CON SDFU increased consistently across d 0, 2, and 6 of incubation (5.49, 5.62, and 6.12, respectively), while in the case of NaL-O there was no further pH increase after d 2.

Table 6 shows the effects of the CLN and DAY interaction on bacterial counts and WSC. The counts of all tested bacteria were higher at d 6 vs. 0 across all bedding cleanliness levels, except for *P. aeruginosa* in SD. Across all CLN levels for *E. coli*, d 2 counts were no different than d 0, except for the SD bedding, but were always lower than d 6. In the case of *K. pneumoniae* and *S. uberis*, there were no differences in counts between d 2 and 0 across all beddings. A similar trend was observed for *S. chromogenes*, except for SDU, which showed a higher count at d 2 vs. 0. Higher counts were observed at d 6 vs. d 2 for *K. pneumoniae* and *S. chromogenes* across all CLN levels. *S. uberis* had higher counts at d 6 vs. d 2 for SD and SDFU but no differences were observed for SDU and SDF. *P. aeruginosa* showed no count differences across incubation days for SD, but d 2 had higher counts than d 0 for SDU and SDFU, but not for SDF. There were higher counts of *P. aeruginosa* at d 6 vs. 2 for SDU, SDF, and SDFU.

At d 0, SDF had higher counts for all bacteria vs. SD and SDU. SDFU had higher counts than SD and SDU for *E. coli* and *K. pneumoniae*; but only higher than SDU for *S. chromogenes* and *P. aeruginosa* at d 0 (Table 6). After 2 d of incubation, the same patterns were observed for *E. coli*, *K. pneumoniae*, and *S. uberis*. In the case of *S. chromogenes*, SDF had higher counts than all other bedding types after 2 d, but there were no differences between the other types. *P. aeruginosa* had higher counts after 2 d for SDF vs. SD and SDU, and SDFU had higher counts than SDU. On the last day of incubation (d 6), all beddings that included feces had higher counts compared to SD and SDU for *E. coli*, *K. pneumoniae*, and *P. aeruginosa*. In the case of *S. uberis* and *S. chromogenes* (Gram-positives), only SDFU had higher counts than the ones in SD and SDU at d 6. Also, there were no count differences between SDF and SDFU for all bacteria tested at d 6, except for *S. uberis* and *S. chromogenes*. It seems that certain urine components stimulated the growth of the Gram-positive bacteria in this study when feces were present after 6 of incubation. SDU had the lowest counts for *E. coli*, *K. pneumoniae*, and *S. chromogenes*. For *P. aeruginosa* and *S. uberis*, both SDU and SD had the lowest counts.

WSC levels were affected by CLN × Day (Table 2). At d 0, SD had a lower WSC concentration than the other bedding types ($\overset{-}{x}$ = 1.70% of DM; Table 6). After 6 d, SD still had the lowest WSC value (0.76% of DM) but SDU (1.32% of DM) also had lower levels than SDF and SDFU ($\overset{-}{x}$ = 2.68). The concentration of WSC after 6 d of incubation was higher for SDFU and SDU but no changes occurred for SD and SDU beddings, relative to d 0. Moreover, beddings treated with NaL-O had a higher WSC concentration compared to CON (2.12 vs. 1.22 ± 0.31% of DM; *p* < 0.001). An effect of CLN was observed for N (% of DM) bedding concentration (Table 2). The highest levels were observed in SDFU (0.64 ± 0.018% of DM), followed by SDF (0.51), and SDU (0.34); SD was the lowest (0.17; *p* < 0.001). In the case of NH_3_-N, we detected the effects of CND and CLN (Table 2). The SDU bedding had the lowest concentration (0.78 vs. $\overset{-}{x}$ = 1.21 ± 0.104% of N; *p* < 0.005). Moreover, all CON beddings had higher NH_3_-N than NaL-O (1.28 vs. 0.91 ± 0.075% of N; *p* < 0.001). Lastly, we observed CND, CLN, and Day effects on bedding NDF levels (Table 2). The bedding with feces ($\overset{-}{x}$ = 69.6% of DM) had much lower values vs. SD and SDU ($\overset{-}{x}$ = 79.6 ± 1.50% of DM; *p* < 0.001). Also, the addition of NaL-O to all beddings reduced NDF levels compared to CON (72.8 vs. 76.4 ± 2.37% of DM). After 6 days of bedding incubation, NDF increased from 73.1 to 76.0 ± 2.37% of DM.

## 3. Discussion

Bedding conditioners are used to reduce the risk of mastitis in lactating cattle by mitigating pathogen loads in stalls [15]. Currently available conditioners rely only on altering pH beyond the optimal range for most environmental bacteria (4.4 to 8.7) [36]. However, certain pathogens, such as *Klebsiella* spp., can survive in vitro at a pH as low as 3 [37]. Consequently, existing bedding conditioner technologies provide only transient pathogen reductions, lasting up to 1 d in organic beddings [11,19]. This short-lived effectiveness highlights the need for more sustainable solutions, especially for sawdust bedding, one of the most commonly used materials on dairy farms. A survey of 325 farms found that 21.1% (68 farms) used sawdust bedding [15]. In these systems, sawdust is reapplied every 4 d to maintain a depth of ~10 cm, while manure and soiled bedding are removed [19]. To achieve prolonged pathogen suppression, more effective and longer-lasting bedding conditioners need to be developed.

In our study, the extent to which NaL-O reduced bacterial counts varied by pathogen, incubation day, and bedding cleanliness. *P. aeruginosa* was the only pathogen consistently inhibited by NaL-O, regardless of these factors. In contrast, the inhibitory effects on *E. coli* and *K. pneumoniae* were reduced in the presence of feces in the bedding, with the magnitude of the reduction being constant across incubation days. Interestingly, in the SDF and SDFU beddings, these two bacteria had higher counts than in SD and SDU beddings from d 0 to 6. It is likely that there were components in the feces that were especially protective for *E. coli* and *K. pneumoniae*. This is supported by the increase in WSC levels observed only in SDF and SDFU over this period. Moreover, bacterial competition for scant nutrients across varying levels of fecal contamination, combined with the stress caused by NaL-O, may have influenced these dynamics, as all tested bacteria species were mixed within the bedding.

For the Gram-positive bacteria *S. uberis* and *S. chromogenes*, NaL-O decreased counts regardless of bedding cleanliness, but its efficacy was lessened as incubation days increased from 0 to 6. The strong antimicrobial activity of NaL-O against these two Gram-positive bacteria aligns with our MIC and MBC results for NaL, which showed that they were the most susceptible species. A reduction in bedding conditioner effectiveness over time has also been reported for other conditioner types. For instance, a lime-based conditioner composed of CaCO_3_, MgCO_3_, Ca(OH)_2_, and Mg(OH)_2_ initially reduced bacterial counts by 0.32 log CFU/g for Gram-negative bacteria and 0.21 log CFU/g for *E. coli* after increasing the pH to approximately 9.5–10.0 within one d. However, its antibacterial effects were reduced over 7 d with continued use [38]. Kuechle et al. [39] showed that the addition of an alkalinizing conditioner reduced coliform, *Klebsiella* spp., and *Streptococci* counts for at least 2 d post-application. In our study, NaL-O maintained its inhibitory effects on all bacteria for up to 6 d. In comparison, Hogan et al. [13] observed a significant reduction in bacterial counts in organic bedding materials after 1 d of using a commercial conditioner containing 93% sodium hydrosulfate. Moreover, Proietto et al. [19] found that a clay-based acidic conditioner reduced total Gram-negative bacteria counts for 2 days in dairy cattle bedding.

The interaction between bedding cleanliness and incubation time was evident for bacterial counts and key bedding chemical properties, as feces not only increased bacterial counts as incubation time increased but also affected chemical properties like pH and WSC (Table 2). The bedding pH was higher as incubation time increased for treatments that included feces only, compared to SD and SDU (Table 5). Notably, a similar pattern was observed for WSC in treatments including feces, with higher values as incubation days were extended. The higher bacterial count in the beddings contaminated with feces influenced certain chemical properties of the beddings, like pH and WSC, most likely through an increase in bacterial metabolization of nutrients [40,41]. Interestingly, an increase in NDF was reported across all beddings as incubation time was extended. The lower NDF content in beddings with feces suggests that more nutrients were available in such beddings considering that the bacteria species tested lack the ability to break down plant fibers significantly [42]. Thus, maintaining clean bedding, especially in regard to fecal matter, is essential for minimizing bacterial load and promoting cow health [43]. A study found that feces in heap recycled manure solids bedding had a higher bacterial load, with 7.1 log CFU/g and 7.2 log CFU/g after 24 h inoculation with *E. coli* and *K. pneumoniae*, respectively, compared to new sand bedding, which had bacterial loads of 1.8 log CFU/g and 2 log CFU/g over 1 to 3 days of incubation [44]. The presence of feces in bedding exacerbates bacterial proliferation. This effect was observed in a study where bedding with manure solids showed a very high population of *Klebsiella* spp. as usage days increased [45]. In the same study, herds using manure solids as bedding generally had dirtier udders, associated with bedding material supporting higher bacterial counts, and compromised milk quality (higher SSC) compared to herds using recycled sand. This was likely due to manure solids retaining more moisture, leading to microbial proliferation and increasing the risk of infection. Kuechle et al. [39] observed that bedding materials with elevated pH levels, such as digested manure solids and recycled sand, supported the growth of *Enterococcus faecium*, whereas materials with lower pH levels, such as shavings, did not promote *E. faecium* growth. This emphasizes the importance of keeping pine-sawdust-based beddings free of feces, as they naturally have a low pH [46]. Our CON white pine sawdust bedding (SD) had a main effect mean pH value of 3.83 (Table 5), and incubation time did not affect it. Consequently, the SD bedding sustained limited bacterial growth compared to SDF and SDFU, and in the case of *P. aeruginosa* it did not sustain any growth across incubation days. On the other hand, feces increase bedding pH and provide nutrients to the pathogenic bacteria tested. This suggests that manure favors bacterial growth regardless of bedding types. Good farming practices (e.g., regularly cleaning or changing bedding) are crucial in keeping bedding hygienic and reducing the concentration of pathogenic bacteria that may come into contact with teat ends and potentially cause intramammary infections.

Understanding the antimicrobial properties of technical lignins and comparing findings across studies is challenging because of variations in methodologies [8], type of technical lignins [47], and microbial species used in experiments [26]. Moreover, many studies lack thorough descriptions of lignin chemical properties, thereby complicating direct comparisons. Lignosulfonates are bio-based surfactants and specialty chemicals, which are generated by breaking the complex lignin network during sulfite pulping of wood [48]. A key advantage is their low cost ($400–1000/metric ton), making them an affordable alternative. Removing impurities such as sugar and ash can potentially enhance lignins’ antimicrobial properties, boosting efficacy and allowing for lower application rates [49]. Furthermore, the United States Food and Drugs Association has deemed magnesium, calcium, ammonium, and sodium lignosulfonates safe for inclusion in animal feed at up to 4%, suggesting that their use as bedding conditioners poses minimal health risks to livestock [50]. Lignosulfonates are generally considered safe, as they are non-toxic and non-irritating [34]. The antimicrobial activity of lignosulfonates is strongly pH-dependent, with greater potency in acidic conditions. For instance, at pH 4 and a 3% concentration, sodium lignosulfonate showed 100% inhibition against several fungi, while magnesium lignosulfonate (MgL) showed variable effectiveness [32]. Sodium lignosulfonate effectively inhibited *Aspergillus amoenus*, *Penicillium solitum*, *Mucor circinelloides*, and *Debaryomyces hansenii* at pH 4 compared to pH 6, suggesting that a lower pH enhances its antimicrobial properties. This pH-dependent behavior is linked to ionization changes in lignosulfonate functional groups, impacting interactions with microbial membranes [51]. Lignosulfonates exert antimicrobial effects by binding bacterial cell walls through electrostatic and hydrophobic interactions, disrupting microbial functions via multiple mechanisms [48]. In addition, their surfactant properties enable interactions with cellular components, particularly lipids and proteins, disrupting microbial cellular functions and negatively impacting cell growth and viability [52,53].

The antibacterial effect of lignosulfonates was reported in a study where sodium lignosulfonate was used as a component of an acidified wood-derived litter combined with formic acid and propionic acid, with the ultimate objective of reducing harmful bacteria like *Clostridium perfringens* and *Enterococcus* spp. in poultry beddings so the risk of necrotic enteritis and other diseases can be reduced in poultry operations [54]. That study showed that the addition of this bedding conditioner improved intestinal health, nutrient absorption, and overall growth performance in poultry. Additionally, adding the bedding conditioner stabilized intestinal flora and reduced the spread of pathogens in crowded environments, offering economic and health benefits for poultry producers. In our study, both NaL and MgL exhibited inhibitory and killing properties against pathogenic bacteria associated with mastitis in cattle, supporting their potential for mitigating pathogenic microbes in animal beddings.

A study showed that lignin extracts from corn stover exhibited antimicrobial activities against Gram-positive bacterial pathogens, including *Listeria monocytogenes* and *Staphylococcus aureus* [26]. While NaL exhibited superior antimicrobial properties compared to AKL and WKL, it is important to consider the effects of Kraft lignins because of their vast abundance [10]. Dong et al. [26] reported that alkali Kraft lignin (Sigma-Aldrich Corp, St. Louis, MO, USA) showed a MIC of 0.01 and 0.0025 μg/mL against Gram-positive bacteria (*L. monocytogenes* and *S. aureus*) and yeast (*Candida lipolytica), respectively*, but was not effective against Gram-negative bacteria (*E. coli* O157 and *Salmonella enterica*). In that study, variations in lignin extraction conditions, such as temperature and residue or solvent ratio, influenced its antioxidant and antimicrobial properties. Consistent with those findings, we observed minimal activity of AKL against Gram-negative bacteria (*P. aeruginosa*). The exact mode of action of Kraft lignins against bacteria remains unclear, though Dizhbite et al. [55] suggested that their antimicrobial effects may stem from the inhibition of key chemical reactions in bacterial cells. This activity could be associated with the high antiradical potential of their methanol-soluble fraction. In addition, AKL shows selective antimicrobial activity, effectively targeting Gram-positive bacteria while lacking efficacy against Gram-negative species [26]. After 24 h of incubation, reductions of 7.07 log CFU/mL for *L. monocytogenes* and 6.77 log CFU/mL for *C. lipolytica* were reported. Antioxidant activity was measured between 1742 ± 930.3 μmol TE/g and 3120 ± 928.9 μmol TE/g. Furthermore, Dong et al. [26] reported an MIC of 0.01 μg/mL for AKL against *S. aureus* but no antibacterial activity against *L. monocytogenes*. In our study, AKL exhibited an antioxidant activity of 10.5 μmol TE/g and contained 219.1 mg/g of total soluble phenolics. The limited antibacterial activity of AKL observed in our study compared to Dong et al. [26] is likely due to differences in bacterial species tested, as the lignin source and methodologies used were similar. The antimicrobial properties of lignins are thought to be largely driven by their phenolic content, as phenolic compounds can disrupt microbial cell walls, denature proteins, and interfere with essential enzyme processes [56]. Their hydroxyl groups interact with microbial membranes, leading to cell leakage and death. However, Núñez-Flores et al. [52] did not observe this relationship in lignosulfonates, suggesting that additional factors may influence their antimicrobial effectiveness. In our study, NaL showed the best antibacterial activity compared to other technical lignins tested. The precise mechanism underlying it remains unclear and will be explored in further studies.

## 4. Materials and Methods

### 4.1. Minimum Inhibitory Concentration (MIC) and Maximum Bactericidal Concentration (MBC) Tests

#### 4.1.1. Bacterial Inoculum Preparation

All animal bacterial pathogens tested in this study were isolated from mastitic cows (Table 7). Two strains per bacterial species were evaluated in this experiment. Strains were grown on trypticase soy agar (TSA) plates for 24 h at 37 °C. For each strain, a single colony was selected and then inoculated into a tube containing 5 mL of tryptic soy broth (TSB), which was then incubated overnight at 37 °C with shaking at 210 rpm. Afterward, for each strain, a 1 mL aliquot was dispensed into a 20 mL Erlenmeyer flask containing 9 mL of TSB, which was then shaken (210 rpm) at 37 °C for an incubation time previously determined for each strain to reach the late log phase. Once each strain reached an optical density of 1.0 at 600 nm (corresponding to 5 × 10^8^ CFU/mL), a 100 µL aliquot was taken and dispensed into 9.9 mL of Muller-Hinton broth (MHB) to obtain an inoculum of 5 × 10^6^ CFU/mL for each strain tested. Inoculum counts were verified by serial dilutions plated on TSA. Inoculated plates were incubated overnight at 37 °C and counted.

#### 4.1.2. Bedding Conditioners (CND)

Table 8 outlines the major chemical properties of the technical lignins evaluated in this study. The technical lignins tested in this screening study were partially selected based on the antimicrobial results obtained by Reyes et al. [49], and include sodium lignosulfonate (NaL; Sappi North America, Inc., Westbrook, ME, USA) [8], magnesium lignosulfonate (MgL; Sappi North America, Inc., Westbrook, ME, USA), washed southern pine softwood kraft lignin (WKL), and alkali kraft lignin (AKL; Sigma-Aldrich Corp, St. Louis, MO, USA). The Fourier transform infrared spectra of the lignosulfonates are available in a previous study [22]. Southern pine softwood kraft lignin was used as the substrate to generate the WKL conditioner. Firstly, the southern pine softwood kraft lignin was delignified at a H factor (quantifying the cumulative effect of temperature and time on delignification process) of about 1600 via the kraft process and precipitated using the Lignoboost process with CO_2_ as the acid [57]. Secondly, the southern pine softwood kraft lignin was dissolved in nanopure water to prepare a 10% solution (*w*/*v*, fresh basis). This solution was then filtered through a Whatman No. 54 paper (Fisher Scientific; Pittsburgh, PA, USA) with the help of a MaximaDry vacuum pump (Fisherbrand; Pittsburgh, PA, USA). The kraft lignin residue was repeatedly washed with nanopure water until the pH of the filtrate reached a value of 6. Lastly, the washed lignin was dried in an Isotemp 281A vacuum oven (Fisherbrand; Pittsburgh, PA, USA) at 40 °C and 50.8 mm Hg until constant weight. The final manually pulverized dried residue is referred to as WKL in this study.

#### 4.1.3. Antimicrobial Assays

The MIC was defined as the lowest concentration of CND that prevented the visible growth of the mastitis-causing bacteria. The macrodilution assay was carried out independently three times (each one in duplicate), following the protocol outlined by the Clinical and Laboratory Standards Institute (CLSI). Stock solutions of the CND (20% *w*/*v*) were prepared in sterile Mueller Hinton Broth (BD, Franklin Lakes, NJ, USA) using 50 mL propylene tubes, which were sonicated for 60 min in an 8510 Series Ultrasonic Cleaning Bath (Emerson, St. Louis, MO, USA) containing water at 40 °C to ensure microbial inactivation of CND with minimal effect on the lignin chemical structure [63]. According to the CND concentrations tested (ranging from 0.25 to 6 mg/mL), different volumes of the stock CND and sterile MHB were pipetted into 15 mL borosilicate glass tubes to produce a 5 mL final medium volume. Moreover, 0.15 mL of dimethyl sulfoxide (DMSO) was added to all tubes to ensure a final concentration of 1% *w*/*v*, which aided in evenly dissolving all CND. In addition, HCl or NaOH was added to achieve a final pH of 6 in the medium, respectively. A 50 µL bacterial inoculum aliquot was added to designated tubes to obtain a final concentration of 5 × 10^4^ CFU/mL for each strain. Afterward, all tubes were incubated overnight at 37 °C with shaking (210 rpm). After this period, MIC was determined and if tubes presented no visible growth, a 100 µL aliquot was taken and plated on TSA. Plates were then incubated at 37 °C for 24 h to determine if there were viable cells and the MBC, defined as the lowest CND concentration that kills 99.9% of the initial bacterial concentration [64]. All MIC and MBC values are reported as mean concentrations (mg/mL ± standard deviation).

### 4.2. Antimicrobial Activity of CND in Bedding Substrates

#### 4.2.1. Bedding Substrates

**Figure 1:**
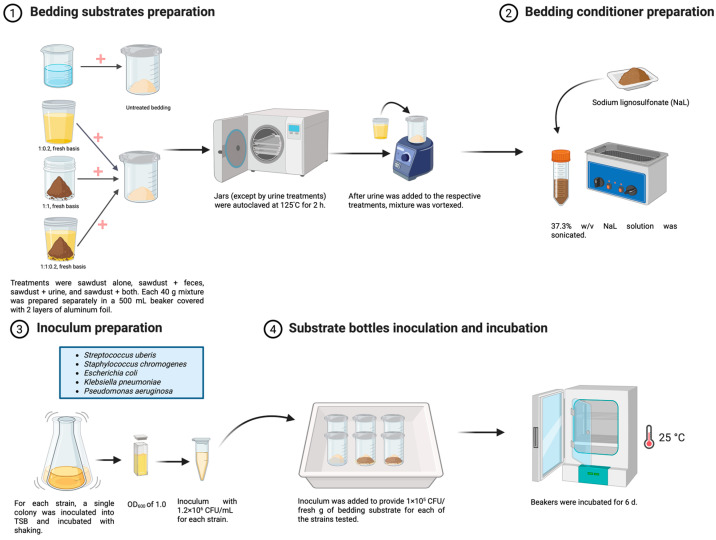
Schematic illustration of the antimicrobial activity of CND in bedding substrates procedures.

The experimental procedures are outlined in Figure 1. Bedding treatments were designed to simulate four levels of cleanliness: sawdust only (SD), sawdust plus feces (SDF), sawdust plus urine (SDU), and sawdust plus feces and urine (SDFU). The bedding cleanliness levels were formulated using the following ratios (fresh basis weights): 1:1 for SDF (sawdust/feces), 1:0.2 for SDU (sawdust/urine), 1:1:0.2 for SDFU (sawdust/feces/urine, respectively), and SD only. The proportions were based on bedding cleanliness data from five Maine dairy farms using sawdust bedding (data unpublished). All experimental units consisted of exactly 40 g (fresh basis) of the bedding substrate mixtures, prepared individually in glass jars (500 mL) following the ratios mentioned above.

Sawdust from Eastern white pine (*Pinus strobus*; 93% DM) was processed by drying at 60 °C to constant weight (72 h) and then ground to pass through the 3 mm screen of a Wiley mill. Fresh feces were obtained from lactating dairy cattle consuming a diet free of antibiotics following an approved protocol by the Institutional Animal Care and Use Committee (IACUC A2020-07-03) of the University of Maine. The collected feces were dried at 60 °C to a constant weight (72 h) and ground using a Wiley mill to pass a 3 mm screen. Free-catch urine samples were collected concurrently with fecal samples and were sterilized by filtering through a 0.22 μm sterile filtration cup using a vacuum pump (Fisher scientific MaximaDry, Pittsburgh, PA, USA). These materials were stored at −25 °C before use.

#### 4.2.2. Bedding Conditioners (CND)

A stock solution of NaL (37.3% *w*/*v*) was prepared in sterile nanopure water using a 50 mL propylene tube, which was sonicated as previously described and modified to produce NaL-O following a proprietary process developed at the University of Maine to maximize its antimicrobial properties. The NaL conditioner was selected because of its promising results in the MIC and MBC tests, which showed that it had the highest inhibitory effect against the mastitis-causing bacteria tested. The control samples (CON) contained only the bedding substrates and no conditioner. For clarity, the procedure used to apply the bedding conditioner in this experiment is outlined in the assay sub-section.

#### 4.2.3. Inoculum Preparation

The following bacterial strains were selected for this assay: *Streptococcus uberis* (BAA-854), *Staphylococcus chromogenes* (KCJ4610), *Escherichia coli* (10.0371), *Klebsiella pneumoniae* (KCJ4749), and *Pseudomonas aeruginosa* (KCJ4633; Table 7). For each strain, a single colony was inoculated into TSB and incubated at 37 °C overnight with shaking (210 rpm). Afterward, a 1 mL aliquot was dispensed into a glass tube containing 9 mL of TSB and incubated until an OD_600_ value of 1.0 was reached (~5 × 10^8^ CFU/mL). Afterwards an aliquot was taken from each strain to prepare a saline-based mixed-inoculum solution containing the five strains, each at a concentration of 1.2 × 10^6^ CFU/mL. Inoculum counts for each strain were verified by preparing serial dilutions plated on CHROMagar mastitis plates (CHROMagar, Saint-Denis, France), which were able to distinguish all strains tested in this experiment. Plates were incubated overnight at 37 °C and counted.

#### 4.2.4. Antimicrobial Activity Assay

The following protocol was followed to (1) normalize the moisture levels for sawdust and feces across all treatments; (2) ensure even mixing of bedding components; (3) eliminate potential confounding effects caused by other bedding microbiota; and (4) translate Mueller–Hinton broth results to an assay using real animal bedding substrate. Nanopure water was added to the dried and ground sawdust and lactating dairy cattle feces to achieve 25 and 86% moisture, respectively, which reflect typical values for such materials. Within the 500 mL glass jars, different amounts of sawdust and feces were added following the proportions stated above (for a total of 40 g, fresh basis). The re-moistened sawdust was thoroughly mixed with a spoon for 3 min and then vortexed. If feces needed to be added, the mixture with sawdust was thoroughly mixed with a spoon for 3 min and then vortexed for a second time. Next, all jars were autoclaved at 125 °C for 2 h to achieve sterile conditions. In the case of bedding treatments that included urine, the filter-sterilized urine was added after the autoclaving step, following the previously mentioned proportions. When urine was added, the mixture was thoroughly mixed with a sterile spoon for 3 min and then vortexed once more.

For the treatments receiving the bedding conditioner, 3.22 mL of the NaL-O stock solution described previously was applied per jar to obtain a final dose in the bedding of 3% (*w*/*w*, fresh basis), and the mixture was thoroughly mixed with a sterile spoon for 3 min and then vortexed once more. Lastly, 3.22 mL of the mixed inoculum solution was added to all jars to provide 1 × 10^5^ CFU/fresh g of bedding substrate for each of the strains tested. Afterward, all the bedding material was mixed thoroughly for 3 min using a sterile spoon, followed by vortexing. The glass jars were then covered with two layers of sterile aluminum foil and incubated at 25 °C for 6 d.

#### 4.2.5. Sampling and pH Analysis

On d 0, 2, and 6, representative samples (5 g, fresh basis) were taken from all jars using sterile tools and then weighed into stomacher bags. Afterward, 45 mL of 0.1% sterile peptone water was added to each bag, which was subsequently blended at 230 rpm for 3 min in a Stomacher blender (Seward Ltd., Worthing, UK). Extracts were filtered using two layers of sterile cheesecloth. The filtrate was collected in 250 mL sterile plastic bottles. Serial 10-fold dilutions were prepared using a sterile saline solution (0.85% saline) and plated on CHROMagar mastitis plates (CHROMagar, France). Plates were incubated at 37 °C overnight to check for counts per bacterial species. The pH of the extract was measured with a calibrated Φ34 Beckman pH meter (Beckman, Brea, CA, USA) fitted with an Accumet Universal pH electrode with an integrated temperature sensor (ThermoFisher Sci., Waltham, MA, USA). The remainder of the filtered extract was acidified to pH 2 using hydrochloric acid (HCl) and frozen at −20 °C for further analysis. On d 6, the rest of the solid samples were frozen at −25 °C for further analysis.

#### 4.2.6. Component Analysis

The solid samples were taken on d 0 and 6 and analyzed for DM at 60 °C (DM; [13]), and then autoclaved at 260 °C for 2 h before being analyzed for neutral detergent fiber (NDF; [65]), nitrogen (N; [66]), and ash. This was performed to ensure operator safety while handling the samples. The acidified liquid samples were analyzed for water-soluble carbohydrates [35] and ammonia-N [67].

### 4.3. Statistical Analysis

In MIC and MBC assays, the determination of MIC and MBC was carried out independently three times in duplicate, and values are reported as mean concentrations (mg/mL ± SD). The antimicrobial activity assay in bedding substrates had a randomized complete block design with a 2 (conditioner, CND) × 4 (bedding cleanliness, CLN) × 3 (incubation time, Day) factorial arrangement of treatments replicated in 5 runs (blocks). The mixed effects model included fixed effects of conditioner, cleanliness, incubation time, and their interaction and the data were analyzed using the GLM procedure of SAS v.94. The random effect was block nested within conditioner type and level of bedding cleanliness. In all models including repeated measures (d 0, 2, and 6), day was the REPEATED statement, and the variance-covariance selected was spatial power because of unequal spacing between sampling days. Means were separated by Tukey’s HSD test, and the SLICE option was used to analyze interactions. Significance was declared at *p* ≤ 0.05.

## 5. Conclusions

Among the technical lignins tested, NaL emerged as the technical lignin with the broadest antibacterial effect against all the mastitic pathogens tested, followed by MgL and AKL. The enhanced effectiveness of NaL-O observed with the evaluated dose underscores its potential as a robust antimicrobial bedding conditioner, warranting further exploration into optimized formulations and application methods. The inherent advantages of NaL-O, including low cost, ready availability, and compatibility with sustainable livestock practices, position it as a viable alternative for controlling mastitis-causing microbes. Overall, bedding cleanliness, the duration of bedding use, and the application of bedding conditioners play crucial roles in inhibiting the growth of mastitis-causing pathogens in animal beddings. Maintaining a clean and sanitary bedding environment is crucial. The addition of a conditioner acts as an extra layer of protection, enhancing the benefits of clean bedding. Beyond its immediate implications, this research lays the foundation for integrating technical lignins into broader strategies to combat microbial threats. Further studies could delve deeper into its long-term impact on microbial resistance patterns and livestock health. Such endeavors will further reinforce the role of technical lignins in advancing sustainable and effective disease control measures in the dairy industry.

## Figures and Tables

**Table 1:** Technical lignins’ minimum inhibitory concentration (DuBois et al., 1956) [35] and minimum bactericidal concentration (MBC) mean values (mg/mL ± SD) against environmental mastitis-causing bacteria.

Bacteria ^1^		Trt ^2^			
		NaL	WKL	AKL	MgL
*S. chromogenes* KCJ4610	MIC	5 ± 0	30 ± 0	2.5 ± 0	10 ± 0
	MBC	7.5 ± 0	60 ± 0	5 ± 0	12.5 ± 0
*S. chromogenes* KCJ4679	MIC	6.25 ± 2.5	>60	16.6 ± 11.5	25 ± 5.77
	MBC	8.75 ± 0.6	-	50 ± 17.3	30 ± 4.8
*S. uberis* BAA-854	MIC	10 ± 0	>60	2.5 ± 0	15 ± 0
	MBC	15 ± 0	>60	2.5 ± 0	40 ± 0
*S. uberis* KCJ145	MIC	5.8 ± 1.4	>60	10 ± 0	23.3 ± 5.77
	MBC	5.8 ± 1.4	-	>60	30 ± 0
*P. aeruginosa* KCJ4633	MIC	35 ± 0	50 ± 0	50 ± 0	60 ± 0
	MBC	>60	>60	>60	>60
*P. aeruginosa* KCJ4686	MIC	16.6 ± 5.77	>60	>60	26.6 ± 11.5
	MBC	20 ± 0	-	-	40 ± 0
*E. coli* 10.0371	MIC	60 ± 0	60 ± 0	>60	30 ± 0
	MBC	60 ± 0	>60	>60	30 ± 0
*E. coli* KCJ3819	MIC	27.5 ± 5.0	>60	>60	20.0 ± 0
	MBC	30 ± 8.2	-	-	56.7 ± 5.77
*K. pneumoniae* KCJ4749	MIC	15 ± 0	n.d	50 ± 0	20 ± 0
	MBC	20 ± 0	>60	>60	20 ± 0
*K. pneumoniae* KCJ4740	MIC	8.33 ± 2.88	n.d	2.5 ± 0	10 ± 0
	MBC	40 ± 0	56.7 ± 1.25	4.37 ± 1.25	10 ± 0

^1^ *Staphylococcus chromogenes*, *Streptococcus uberis*, *Pseudomonas aeruginosa*, *Escherichia coli*, *Klebsiella pneumoniae*. ^2^ Mean ± standard deviation, - = not detectable due to high MIC, n.d = not determined due to technical lignin turbidity.

**Table 2:** Statistical analysis (*p*-values) of the interaction effects for bedding conditioners (CND), bedding cleanliness (CLN), and incubation days (DAY) on bedding chemical properties and environmental mastitis pathogens counts ^1,2^.

Item	*p*-Value						
	CND	CLN	DAY	CND × CLN	CND × DAY	CLN × DAY	CND × CLN × DAY
**Bedding chemical composition**							
DM, %	0.936	<0.001	0.662	0.859	0.520	0.137	0.254
OM, % of DM	<0.001	<0.001	0.649	0.007	0.032	0.560	0.548
WSC, % of DM	<0.001	<0.001	0.007	0.658	0.860	0.011	0.739
N, % of DM	0.816	<0.001	0.102	0.762	0.201	0.639	0.659
NH_3_-N, % of total N	0.002	0.004	0.271	0.892	0.944	0.362	0.779
NDF, % of DM	0.002	<0.001	0.008	0.699	0.269	0.499	0.915
pH	0.102	<0.001	0.002	0.003	0.002	<0.001	0.005
**Pathogen counts (log CFU/fresh g)**							
*Escherichia coli* 10.0371	<0.001	<0.001	<0.001	<0.001	0.228	<0.001	0.959
*Streptococcus uberis* BAA-854	<0.001	<0.001	<0.001	0.165	0.005	<0.001	0.179
*Staphylococcus chromogenes*, KCJ4610	<0.001	<0.001	<0.001	0.197	0.002	<0.001	0.082
*Pseudomonas aeruginosa*, KCJ4633	<0.001	<0.001	<0.001	0.549	0.114	<0.001	0.471
*Klebsiella pneumoniae*, KCJ4749	<0.001	<0.001	<0.001	<0.001	0.686	<0.001	0.987

^1^ CND = Effect of bedding conditioner application; CLN = Effect of bedding cleanliness level; DAY = Effect of incubation days. ^2^ DM = Dry matter; OM = Organic matter; WSC = Water soluble carbohydrates; N = Nitrogen; NH_3_-N = Ammonia nitrogen; NDF = Neutral detergent fiber.

**Table 3:** Effect of bedding conditioners (CND) and incubation days (DAY) on bedding chemical properties and environmental mastitis pathogens counts ^1^.

Item	Bedding Conditioner		SEM
	CON	NaL-O	
**Pathogen counts (log CFU/fresh g of substrate)**			
*Staphylococcus chromogenes* KCJ4610			0.094
d 0	3.38 ^A, y^	2.41 ^B, z^	
d 2	3.50 ^A, y^	2.75 ^B, y^	
d 6	3.95 ^A, x^	3.52 ^B, x^	
*Streptococcus uberis* BAA-854			0.154
d 0	3.02 ^A, y^	1.72 ^B, z^	
d 2	3.19 ^A, y^	2.11 ^B, y^	
d 6	3.69 ^A, x^	3.19 ^B, x^	
**Chemical properties**			
OM ^2^ (% of DM)			0.227
d 0	98.1 ^A^	97.7 ^B, x^	
d 6	98.4 ^A^	97.3 ^B, y^	

^x,y,z^ Means with different lowercase superscripts within a column are significantly different (*p* ≤ 0.05). ^A,B^ Means with different uppercase superscripts within a row are significantly different (*p* ≤ 0.05). ^1^ CON = Untreated bedding; NaL-O = Modified sodium lignosulfonate applied at 3% (*w*/*w*, fresh basis). ^2^ OM = Organic matter; DM = Dry matter.

**Table 4:** Effect of bedding conditioners (CND) and bedding cleanliness on bedding chemical properties and environmental mastitis pathogens counts ^1,2^.

Item	Bedding Cleanliness				SEM
	SD	SDU	SDF	SDFU	
**Pathogen counts (log CFU/fresh g of substrate)**					
*Escherichia coli* 10.0371					0.032
CON	4.07 ^B, x^	3.88 ^C, x^	5.09 ^A, x^	5.05 ^A, x^	
NaL-O	3.76 ^C, y^	3.48 ^D, y^	4.98 ^A, y^	4.89 ^B, y^	
*Klebsiella pneumoniae* KCJ4749					0.018
CON	4.20 ^B, x^	4.01 ^C, x^	5.13 ^A, x^	5.10 ^A, x^	
NaL-O	3.94 ^B, y^	3.68 ^C, y^	5.01 ^A, y^	4.99 ^A, y^	
**Chemical properties**					
OM ^3^ (% of DM)					0.27
CON	98.8 ^A^	98.9 ^A^	98.0 ^B, x^	97.3 ^C, x^	
NaL-O	98.9 ^A^	98.3 ^B^	96.6 ^C, y^	96.3 ^C, y^	

^x,y^ Means with different lowercase superscripts within a column are significantly different (*p* ≤ 0.05). ^A–D^ Means with different uppercase superscripts within a row are significantly different (*p* ≤ 0.05). ^1^ Bedding cleanliness: SD = Sawdust only; SDU = Sawdust plus urine (1:0.2 ratio, fresh basis); SDF = Sawdust plus feces (1:1 ratio, fresh basis); SDFU = Sawdust plus feces and urine (1:1:0.2 ratio, fresh basis, respectively). ^2^ CON = Untreated bedding; NaL-O = Modified sodium lignosulfonate applied at 3% (*w*/*w*, fresh basis). ^3^ OM = Organic matter; DM = Dry matter.

**Table 5:** Effect of bedding cleanliness (CLN), bedding conditioners (CND), and incubation days (DAY) on bedding pH ^1,2^.

Item	Incubation Days			SEM
	0	2	6	
*pH*				0.103
SD				
NaL-O	4.11	4.10	4.03	
CON	3.87	3.85	3.78	
SDU				
NaL-O	4.58 ^A^	4.49 ^B^	4.42 ^B^	
CON	4.50 ^A^	4.41 ^B^	4.32 ^B^	
SDF				
NaL-O	5.03 ^B^	5.11 ^AB^	5.19 ^A, n^	
CON	5.14 ^C^	5.27 ^B^	5.54 ^A, m^	
SDFU				
NaL-O	5.03 ^B, y^	5.19 ^A, y^	5.23 ^A, y^	
CON	5.49 ^C, x^	5.62 ^B, x^	6.12 ^A, x^	

^x,y;m,n;^ Means with different lowercase superscripts within a column are significantly different (*p* ≤ 0.05). x,y applies only for SDFU; m,n apply only for SDF. ^A–C^ Means with different uppercase superscripts within a row are significantly different (*p* ≤ 0.05). ^1^ Bedding cleanliness: SD = Sawdust only; SDU = Sawdust plus urine (1:0.2 ratio, fresh basis); SDF = Sawdust plus feces (1:1 ratio, fresh basis); SDFU = Sawdust plus feces and urine (1:1:0.2 ratio, fresh basis, respectively). ^2^ CON = Untreated bedding; NaL-O = Modified sodium lignosulfonate applied at 3% (*w*/*w*, fresh basis).

**Table 6:** Effect of bedding cleanliness and incubation days (DAY) on bedding chemical properties and environmental mastitis pathogens counts ^1^.

Item	Bedding Cleanliness				SEM
	SD	SDU	SDF	SDFU	
**Pathogen counts (log CFU/fresh g)**					
*Escherichia coli* 10.0371					0.033
d 0	3.88 ^C, y^	3.62 ^D, y^	4.37 ^A, y^	4.26 ^B, y^	
d 2	3.79 ^C, z^	3.64 ^D, y^	4.38 ^A, y^	4.28 ^B, y^	
d 6	4.06 ^B, x^	3.79 ^C, x^	6.36 ^A, x^	6.37 ^A, x^	
*Klebsiella pneumoniae* KCJ4749					0.019
d 0	4.01 ^B, y^	3.77 ^C, y^	4.41 ^A, y^	4.36 ^A, y^	
d 2	4.00 ^B, y^	3.81 ^C, y^	4.41 ^A, y^	4.38 ^A, y^	
d 6	4.19 ^B, x^	3.95 ^C, x^	6.39 ^A, x^	6.40 ^A, x^	
*Streptococcus uberis* BAA-854					0.197
d 0	2.10 ^B, y^	2.31 ^B, y^	3.04 ^A, y^	2.05 ^B, y^	
d 2	2.24 ^B, y^	2.72 ^B, xy^	3.22 ^A, xy^	2.45 ^B, y^	
d 6	2.81 ^C, x^	3.13 ^BC, x^	3.59 ^B, x^	4.23 ^A, x^	
*Staphylococcus chromogenes* KCJ4610					0.119
d 0	2.95 ^B, y^	2.49 ^C, z^	3.26 ^A, y^	2.86 ^B, y^	
d 2	3.09 ^BC, y^	3.00 ^C, y^	3.33 ^A, y^	3.07 ^BC, y^	
d 6	3.53 ^BC, x^	3.34 ^C, x^	3.69 ^B, x^	4.37 ^A, x^	
*Pseudomonas aeruginosa* KCJ4633					0.244
d 0	2.06 ^B^	0.56 ^C, z^	2.70 ^A, y^	1.87 ^B, z^	
d 2	2.13 ^BC^	1.71 ^C, y^	2.72 ^A, y^	2.36 ^AB, y^	
d 6	2.42 ^B^	2.70 ^B, x^	3.42 ^A, x^	3.89 ^A, x^	
**Chemical properties**					
WSC (% of DM)					0.283
d 0	0.82 ^B^	1.43 ^A^	1.74 ^A, y^	1.94 ^A, y^	
d 6	0.76 ^C^	1.32 ^B^	2.75 ^A, x^	2.61 ^A, x^	

^x,y,z^ Means with different lowercase superscripts within a column are significantly different (*p* ≤ 0.05). ^A–D^ Means with different uppercase superscripts within a row are significantly different (*p* ≤ 0.05). ^1^ Bedding cleanliness: SD = Sawdust only; SDU = Sawdust plus urine (1:0.2 ratio, fresh basis); SDF = Sawdust plus feces (1:1 ratio, fresh basis); SDFU = Sawdust plus feces and urine (1:1:0.2 ratio, fresh basis, respectively).

**Table 7:** Bacterial strains and sources.

Microorganism Name (Genus, Species, Strain Name)	Source
*Streptococcus uberis* BAA-854	ATCC
*Streptococcus uberis* KCJ145	University of Florida
*Staphylococcus chromogenes* KCJ4610	University of Florida
*Staphylococcus chromogenes* KCJ4679	University of Florida
*Escherichia coli* 10.0371	Penn State University
*Escherichia coli* KCJ3819	University of Florida
*Klebsiella pneumoniae* KCJ4749	University of Florida
*Klebsiella pneumoniae* KCJ4740	University of Florida
*Pseudmonas aeruginosa* KCJ4633	University of Florida
*Pseudmonas aeruginosa* KCJ4686	University of Florida

**Table 8:** Chemical composition of technical lignins.

Lignin ^1^	Total Soluble Phenolics ^2^	ORAC ^3^ (Mmol Trolox Equivalent (TE)/g DM)	DPPH Scavenging Effect ^4^	WSC ^5^	Ash ^6^	Magnesium ^7^	Sodium	Sulfur
	(mg/g DM)			% of DM				
AKL	219.1	10.53	−4.8	18.05	19.1	0.02	6.86	4.80
WKL	322.2	728.1	74.0	0.11	0.26	0.01	0.04	1.51
NaL	184.3	12.1	14.2	22.8	33.9	0.05	12.8	8.01
MgL	142.5	10.1	10.5	15.7	13.6	6.21	0.04	8.25
Pooled SD	9.14	34.08	12.7	0.45	0.27	0.034	0.084	0.142

^1^ AKL = Alkali Kraft lignin, WKL = Southern pine softwood Kraft lignin; Delignified at a H factor of about 1600 via the Kraft process and precipitated using the Lignoboost process with CO_2_ as the acid [57], NaL = Sodium lignosulfonate, and MgL = Magnesium lignosulfonate lignin. ^2^ Method adapted from Singleton and Rossi [58]. ^3^ Hydrophilic and lipophilic oxygen radical absorbance capacity (ORAC). LBKL were tested by lipophilic ORAC, and AKL, NaL, and MgL were tested by hydrophilic ORAC [26]. ^4^ Method adapted from Wu et al. [59] and AOAC 2012.04. [60]. DPPH = 2,2-diphenyl-1-picrylhydrazyl. ^5^ Water soluble carbohydrates, [35]. ^6^ Method adapted from FAO [61]. ^7^ Method adapted from Beliciu et al. [62].

## Data Availability

The original contributions presented in this study are included in the article. Further inquiries can be directed to the corresponding authors.
